# Metastatic Rectal Small Cell Carcinoma: A Case Report

**DOI:** 10.7759/cureus.9340

**Published:** 2020-07-22

**Authors:** Haisam Abid, Amrat Kumar, Anush Patel

**Affiliations:** 1 Internal Medicine, Bassett Medical Center, Cooperstown, USA; 2 Hematology / Oncology, Bassett Medical Center, Cooperstown, USA

**Keywords:** extrapulmonary small cell carcinoma, chemotherapy, rectal carcinoma

## Abstract

We report a case of metastatic small cell carcinoma presenting as a rectal mass in an 80-year-old male with a history of change in bowel movement and rectal pain for six months. A computed tomography (CT) scan of the abdomen and pelvis was done, which showed a large rectal mass with many liver metastases. He had a diagnostic colonoscopy, which showed a large obstructing rectal mass, and the biopsy result came back as small cell carcinoma. He underwent palliative diverting colostomy without complications. Initially, there was a plan to treat the patient with systemic chemotherapy with etoposide and carboplatin, but given the acute kidney injury, there was a delay in treatment. After the first cycle of chemotherapy, the patient had severe nausea and vomiting. After a discussion with the patient and his family, he decided on hospice care and passed away in a few weeks.

## Introduction

Small cell carcinomas (SCC) are malignancies that originate from neuroendocrine cells. Most commonly, they originate in the lungs but can involve other sites as well, such as breast, thymus, skin, kidney, ovary, uterus, urinary bladder, and pancreas, and are known as extra-pulmonary small cell carcinoma (EPSCC) [1]. Rectal SCC is a very rare tumor comprising less than 1% of all colorectal malignancies [2]. It is an aggressive neoplasm, which usually presents with widespread metastases and carries a poor prognosis [3]. We report a case of metastatic small cell carcinoma presenting as a rectal mass.

## Case presentation

**Figure 1:**
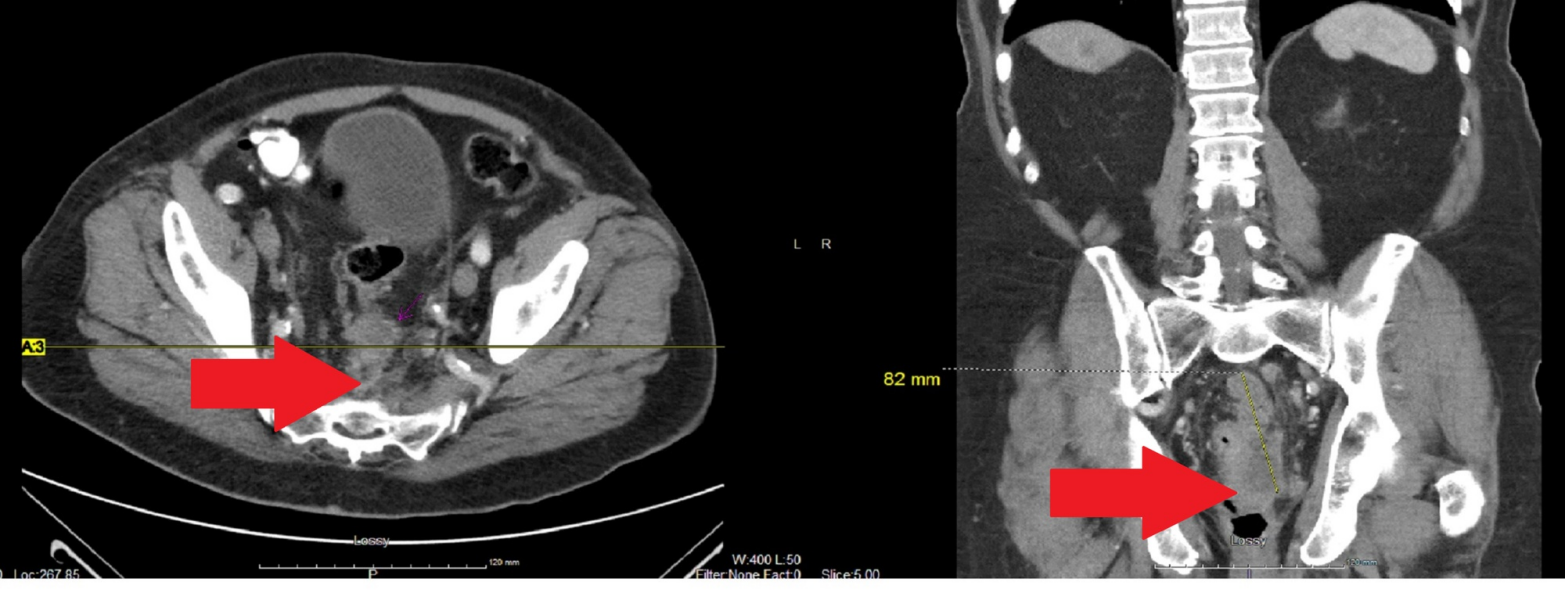
Computed tomography (CT) scan showing a rectal mass

An 80-year-old male with a medical history of atrial fibrillation and aortic valve replacement on long-term anticoagulation presented to the clinic with abdominal symptoms of bloating and rectal pressure. He also complained of nausea, poor appetite, and a 10-pound weight loss in the last three weeks. He had noticed a change in his bowel movement, from diarrhea to small, hard, pebble-like bowel movements. He did not have any rectal bleeding. On examination, he was hemodynamically stable and the abdominal examination was unremarkable except for dullness to percussion in the left flank and the left lower quadrant. Digital rectal examination (DRE) showed a rectal mass and mild anal stricture. The abdominal X-ray did not reveal any bowel obstruction. Computed tomography (CT) abdomen and pelvis showed a rectal mass with surrounding lymphadenopathy (Figure 1).

**Figure 2:**
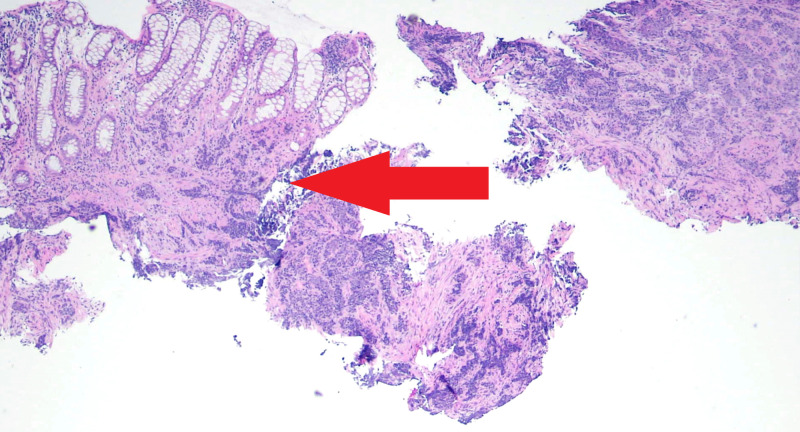
Histopathology showing superficial portions of the mucosa involved by a tumor composed of irregular, solid nests and aggregates of intermediate size, nuclear hyperchromasia, scant cytoplasm, and abundant apoptotic bodies and mitotic figures, consistent with small cell carcinoma

The patient was admitted to the hospital and underwent diagnostic colonoscopy, which showed an obstructing rectal mass. Histopathology showed superficial portions of mucosa involved by a tumor composed of irregular, solid nests and aggregates of intermediate size, nuclear hyperchromasia, scant cytoplasm, and abundant apoptotic bodies and mitotic figures consistent with small cell carcinoma (Figure 2).

There was some apprehension from the patient’s perspective to start him on systemic chemotherapy, though it was not initiated, waiting for the biopsy report. The patient underwent palliative diverting colostomy without any complications and was later discharged with a plan to start palliative chemotherapy. He did not tolerate chemotherapy and opted for hospice care and passed away in a few weeks.

## Discussion

Extra-pulmonary small cell carcinoma (EPSCC) was first described by Kennedy and Duguid in 1930. It has been increasingly recognized as a distinct entity from SCC of the lungs [1]. Neuroendocrine tumors include well-differentiated carcinoid tumors and aggressive neuroendocrine carcinomas, which are also known as SCC [2]. SCCs are the most poorly differentiated tumors of neuroendocrine cells [4]. Neuroendocrine cells are present in various organs, including the gastrointestinal tract (GIT), though rectal small cell carcinoma accounts for less than 1% of colorectal cancers [5].

SCC of the rectum usually presents as a large ulcerative rectal mass. The most common symptoms associated with it include a change in bowel movements, rectal pain, and blood in stools. The pathogenesis and risk factors associated with rectal SCC are unknown [2]. Some studies report that SCC may arise from pluripotent neuroendocrine stem cells or may arise from cells in pre-existing adenomas. There have been a few cases reported in the literature, which shows an association of SCC with tubulovillous adenomas [2-3].

The histopathological features of EPSCC are identical to pulmonary small cell carcinoma. These features include the presence of small, round, or spindle-shaped cells, with intensely hyperchromatic nuclei, scant cytoplasm, and frequent mitoses, and at least two neuroendocrine markers (neuron-specific enolase, chromogranin, synaptophysin) must be positive for a definite diagnosis of SCC. Among these neuroendocrine markers, synaptophysin is the most reliable marker [6].

A retrospective study conducted in the United States on 64 patients showed that the most common location for EPSCC is the colon and rectum. The median age of onset is 55 years, with male predominance. This remains an aggressive disease with a median survival of weeks without treatment and could be six to 12 months in patients receiving treatment [7]. Another retrospective study conducted in Korea among 24 patients showed that the median overall survival rate is 15.3 months and the three-year survival rate is 30% [1]. A meta-analysis by Brenner et al. noticed the pattern of metastasis is liver, followed by lymph node, and then bone metastasis, suggesting hematogenous spread [7]. Our patient had multiple lesions in the liver.

The optimal treatment for rectal SCC remains unknown given the rarity of this tumor [3]. The treatment approach is extrapolated from experiences in SCC of lungs. Spiliopoulou et al. divided SCC of the rectum into two major groups, limited disease or extensive disease, based on whether the disease extent could be covered by an acceptable radiotherapy portal. The treatment options for extensive disease are systemic chemotherapy or supportive care. The approach for limited disease is not as clear; some experts suggest local treatment using surgery or radiotherapy while others suggest multimodality approaches or chemotherapy alone [1-8]. In another study comprising 81 patients with EPSCC, the majority of patients presented with limited disease, and the combination of chemotherapy and radiation therapy was observed to be as effective as surgery. It has also been suggested that local control of extra-pulmonary small cell carcinoma of the rectum may be achieved with multidrug chemotherapy and radiation therapy, without the requirement for radical surgery [8]. Surgery should only be performed on small, localized tumors and chemo-radiation should be administered in patients presenting with advanced stage [2]. In the literature review, the incidence of brain metastases is low, so prophylactic cranial irradiation is not routinely recommended [9-10].

## Conclusions

Metastatic rectal SCC is an extremely rare type of colorectal malignancy. It is most likely to metastasize to the liver, lung, bone, and lymph nodes. It carries a poor prognosis due to its aggressive tumor biology. Due to the rarity of the tumor, there is limited data regarding the optimal treatment. This case also places emphasis on obtaining accurate pathology prior to proceeding with the treatment plan, as that can change treatment and prognosis.
